# Opposite pattern of transcranial direct current stimulation effects in middle-aged and older adults: Behavioral and neurophysiological evidence

**DOI:** 10.3389/fnagi.2023.1087749

**Published:** 2023-01-25

**Authors:** Chiara Bagattini, Susana Cid-Fernández, Martina Bulgari, Carlo Miniussi, Marta Bortoletto

**Affiliations:** ^1^Neurophysiology Lab, IRCCS Istituto Centro San Giovanni di Dio Fatebenefratelli, Brescia, Italy; ^2^Section of Neurosurgery, Department of Neuroscience Biomedicine and Movement Sciences, University of Verona, Verona, Italy; ^3^Department of Developmental and Educational Psychology, University of Santiago de Compostela, Santiago de Compostela, Galicia, Spain; ^4^Center for Mind/Brain Sciences (CIMeC), University of Trento, Rovereto, Italy

**Keywords:** transcranial direct current stimulation, aging, episodic memory, event-related potentials, dorsolateral prefrontal cortex

## Abstract

**Introduction:**

Episodic memory (EM) exhibits an age-related decline, with overall increased impairment after the age of 65. The application of transcranial direct current stimulation (tDCS) to ameliorate cognitive decline in ageing has been extensively investigated, but its efficacy has been reported with mixed results. In this study, we aimed to assess whether age contributes to interindividual variability in tDCS efficacy.

**Methods:**

Thirty-eight healthy adults between 50 and 81 years old received anodal tDCS over the left prefrontal cortex during images encoding and then performed an EM recognition task while event-related potentials (ERPs) were recorded.

**Results:**

Our results showed an opposite pattern of effect between middle-aged (50–64 years) and older (65–81 years) adults. Specifically, performance in the recognition task after tDCS was enhanced in older adults and was worsened in middle-aged adults. Moreover, ERPs acquired during the recognition task showed that two EM components related to familiarity and post-retrieval monitoring, i.e., Early Frontal and Late Frontal Old-New effects, respectively, were significantly reduced in middle-aged adults after anodal tDCS.

**Discussion:**

These results support an age-dependent effect of prefrontal tDCS on EM processes and its underlying electrophysiological substrate, with opposing modulatory trajectories along the aging lifespan.

## 1. Introduction

Aging is accompanied by a progressive decline in brain structure, physiology, and function, with increased risks of developing various neurological and cognitive disorders, e.g., dementia and Alzheimer’s disease (Blinkouskaya et al., 2021). Cognitive functioning has relevant practical and direct impacts on older individuals since it plays a crucial role in their quality of life. Therefore, studies into whether there are strategies to counteract cognitive decline in aging are of utmost importance.

Among cognitive domains, episodic memory (EM) exhibits the largest degree of age-related impairment, with an augmented decline usually observed after the age of 65 (Rönnlund et al., 2005; Guillaume et al., 2009; Vestergren and Nilsson, 2011). EM is a part of the explicit long-term memory that stores information about personal life, such as temporally dated episodes or events (Tulving, 1984). Therefore, it enables a person to remember directly experienced events, making it possible to be consciously aware of a well-contextualized past experience (Tulving, 1993).

Anodal transcranial direct current stimulation (atDCS) has attracted substantial attention in recent years as a tool to improve memory performance, as well as other cognitive functions (see Goldthorpe et al., 2020; Sandrini et al., 2020; Huo et al., 2021; Indahlastari et al., 2021; Sanches et al., 2021; Siegert et al., 2021, for recent reviews). In fact, there is evidence in young participants demonstrating that its application can modulate ERPs elicited in an EM task (Lu et al., 2015). tDCS consists of the application of a weak electrical current (typically 1–3 mA) through two or more electrodes placed over the scalp that reach the cortex and modulate neuronal transmembrane potentials (Purpura and McMurtry, 1965; Nitsche and Paulus, 2000). Despite the simplistic view that atDCS increases neuronal excitability and enhances behavioral performance (whereas cathodal tDCS decreases neuronal excitability and worsens behavior), the scientific literature clearly demonstrates that tDCS does not always lead to such linear neuropsychological and behavioral outcomes (Fertonani and Miniussi, 2017).

The results regarding the cognitive effects of tDCS in aging are mixed, as the administration of atDCS has not consistently resulted in the enhancement of cognitive functions in older individuals. Some tDCS studies have reported memory improvement in healthy older adults when stimulating the prefrontal cortex (Manenti et al., 2013, 2017; Sandrini et al., 2014, 2016, 2019; Medvedeva et al., 2019; Huo et al., 2020) or the left temporoparietal cortex (Antonenko et al., 2019). On the other hand, other studies applying tDCS over the prefrontal cortex reported no effects or even a worsening in aging (Leach et al., 2016, 2019; Peter et al., 2019; Habich et al., 2020a,b). Therefore, it is clear that brain responsiveness to tDCS shows high inter-individual variability.

Several studies have identified age as a key modulating factor of neural and behavioral effects of tDCS (e.g., Antonenko et al., 2016, 2018; Fiori et al., 2017; Martin et al., 2017). Considering the evidence for age-related structural and functional reorganization, tDCS may operate, at different ages, upon different neural processes (Antonenko et al., 2019). Importantly, these studies suggest that results obtained in an age group may not be transferable to another age group. However, the current literature does not allow us to understand the role of age as a moderating factor of the interindividual variability of tDCS effects within the aged population itself. First, the available literature considered the older population in a wide range of ages covering over 30 years with little or no investigation of the large inter-individual variability within this range [e.g., in Antonenko et al. (2019) authors included participants from 50 to 80 years; in Fiori et al. (2017) and Leach et al. (2019) 60–80 years; in Leach et al. (2016) 60–90 years]. Second, the studies on age-mediated effects of tDCS have focused exclusively on the comparison of older (usually in a range from 50 years and older) with younger (aged 18–35) adults. Brain responsiveness to tDCS among older individuals may even vary more than among younger individuals due to considerable age-related inter-individual differences in cognitive decline and brain structure alterations in this population (Craik et al., 1994; Mungas et al., 2010; Reuter-Lorenz and Park, 2014; Antonenko et al., 2019). Additionally, susceptibility to plasticity-inducing mechanisms is affected in the older adults (Brosnan et al., 2018; Antonenko et al., 2019), and consequently, the brain response to tDCS may be altered. Hence, the heterogeneity of findings on the effects of tDCS on memory in aging further highlights the complexity of the underlying mechanisms (Antonenko et al., 2019) and requires monitoring for age-related inter-individual differences.

In this scenario, middle-aged adults are of major interest, as it may be the stage when the first changes in brain activity occur even in the absence of behavioral decline (Guillaume et al., 2009). To the best of our knowledge, only one study compared, in *a posteriori* analysis, middle-aged (<63 years) and older (>63 years) adults, pointing to a larger reduction in GABA concentrations after tDCS over the sensorimotor region in middle-aged adults. However, no behavioral data that can distinguish group performance were collected (Antonenko et al., 2017).

In sum, the understanding of tDCS effects on the aged brain is still inadequate (Antonenko et al., 2019), although older individuals may be among the primary targets of interventions. Therefore, more studies comparing middle-aged with older adults would help to clarify how tDCS effects on EM may change depending on the subject age.

The mechanisms involved in EM have been extensively studied using the event-related potentials (ERPs) technique, identifying three main components: (1) the Early Frontal effect (EF), a positive component that usually appears at ~300–500 ms and is maximal at frontal locations, thought to reflect familiarity-based recognition processes (Curran, 2000; Rugg and Curran, 2007); (2) the Parietal Effect (P), a subsequent positive component that appears in the ~400–800 ms interval at left parietal regions and is thought to index recollection-based recognition processes (Rugg and Curran, 2007); and (3) the Late Frontal effect (LF), a later positive ERP component that has been associated with post-retrieval monitoring and evaluation processes, and is likely linked to executive function of the prefrontal cortex (Friedman and Johnson, 2000; Rugg, 2004; Hayama et al., 2008). To our knowledge, no studies have evaluated how tDCS modulates ERP components associated with EM in a healthy aged population.

In the present study, we aimed to delineate the trajectories of atDCS effects applied over the left dorsolateral prefrontal cortex (DLPFC) during the encoding phase of an EM task in different aging stages. Two groups of adults were compared: middle-aged (age ranging from 50 to 64 years) vs. older (65 to 81 years) adults. Behavioral and ERP measures were collected in the recognition phase, yielding neural evidence about how tDCS affects the different processes involved in EM retrieval. We expected to find different neural and behavioral effects between age groups. Elucidating the behavioral and neural effects induced by tDCS along the healthy aging continuum may help the development of effective tDCS protocols specifically tailored to the aging brain, and to age-related neurodegeneration.

## 2. Materials and methods

### 2.1. Participants

Nineteen middle-aged (10 females; age: mean = 57.11 years; SD = 0.95; min-max: 50–64) and 19 older (11 females; age: mean = 71.63 years, SD = 1.02; min–max: 65–81) healthy volunteers took part in the experiment. All participants were right-handed, had normal or corrected-to-normal vision and had no history of psychiatric or neurological disorders. Before being enrolled in the experiment, subjects visited the laboratory for a preliminary testing session to complete a comprehensive neuropsychological assessment (refer to Table 1 for the whole list of the administered tests) to confirm the absence of any cognitive deficits. A pathological score on one or more neuropsychological tests was considered an exclusion criterion. The demographic, clinical, and neuropsychological results of the two groups of participants are reported in Table 1. Participants gave written informed consent prior to their participation in the study. All the procedures conformed to the Declaration of Helsinki for research involving human subjects and were approved by the Ethics Committee of the IRCCS Centro San Giovanni di Dio Fatebenefratelli (Brescia, Italy).

**Table 1:** Mean values and standard deviations (in parentheses) of the demographical, clinical and neuropsychological measures of the two groups of participants.

	Middle-aged adults (50–64 years-old)	Older adults (65–85 years-old)	*Value of p*
Demographic and clinical characteristics			
Gender (males/females)	9/10	8/11	0.744
Age (years)	57.11 (4.16)	71.63 (4.44)	**0.000**
Education (years)	13.05 (2.93)	9.47 (3.27)	**0.001**
Stimulation order (anodal/sham)	10/9	8/11	0.516
MMSE	28.61 (1.93)	27.89 (1.75)	0.254
GDS	4.89 (5.75)	5.65 (3.67)	0.648
Neuropsychological assessment			
*Memory*			
RAVLT recall, immediate	46.65 (7.81)	50.44 (7.35)	0.144
RAVLT recall, delayed	10.40 (2.31)	11.39 (3.14)	0.284
Episodic memory	15.42 (3.16)	16.26 (3.86)	0.476
ROCF recall	18.97 (4.76)	19.74 (6.54)	0.690
Digit span forward	5.70 (0.92)	5.72 (0.81)	0.950
Spatial span	5.23 (0.73)	5.26 (0.64)	0.905
*Praxia*			
ROCF copy	34.16 (1.10)	34.02 (2.74)	0.852
*Attentive and executive functions*			
TMT A	24.16 (7.78)	26.18 (19.46)	0.679
TMT B	73.79 (35.01)	51.47 (55.63)	0.154
*Language*			
Verbal fluency, phonemic	40.68 (8.69)	40.59 (10.91)	0.977
Verbal fluency, semantic	48.94 (7.92)	46.93 (6.90)	0.453
*Abstract reasoning*			
RCPM	33.11 (1.88)	33.59 (4.21)	0.654

*p*-values obtained from the *t*-test or Chi-squared test used to compare both age groups are reported (bold value of *p*s indicate significant differences between groups). MMSE, Mini Mental State Examination; GDS, Geriatric Depressive Scale; RAVLT, Rey Auditory Verbal Learning Test; ROCF, Rey–Osterrieth Complex Figure; TMT, Trail Making Test; RCPM, Raven Colored Progressive Matrices.

### 2.2. Experimental procedure

**Figure 1:**
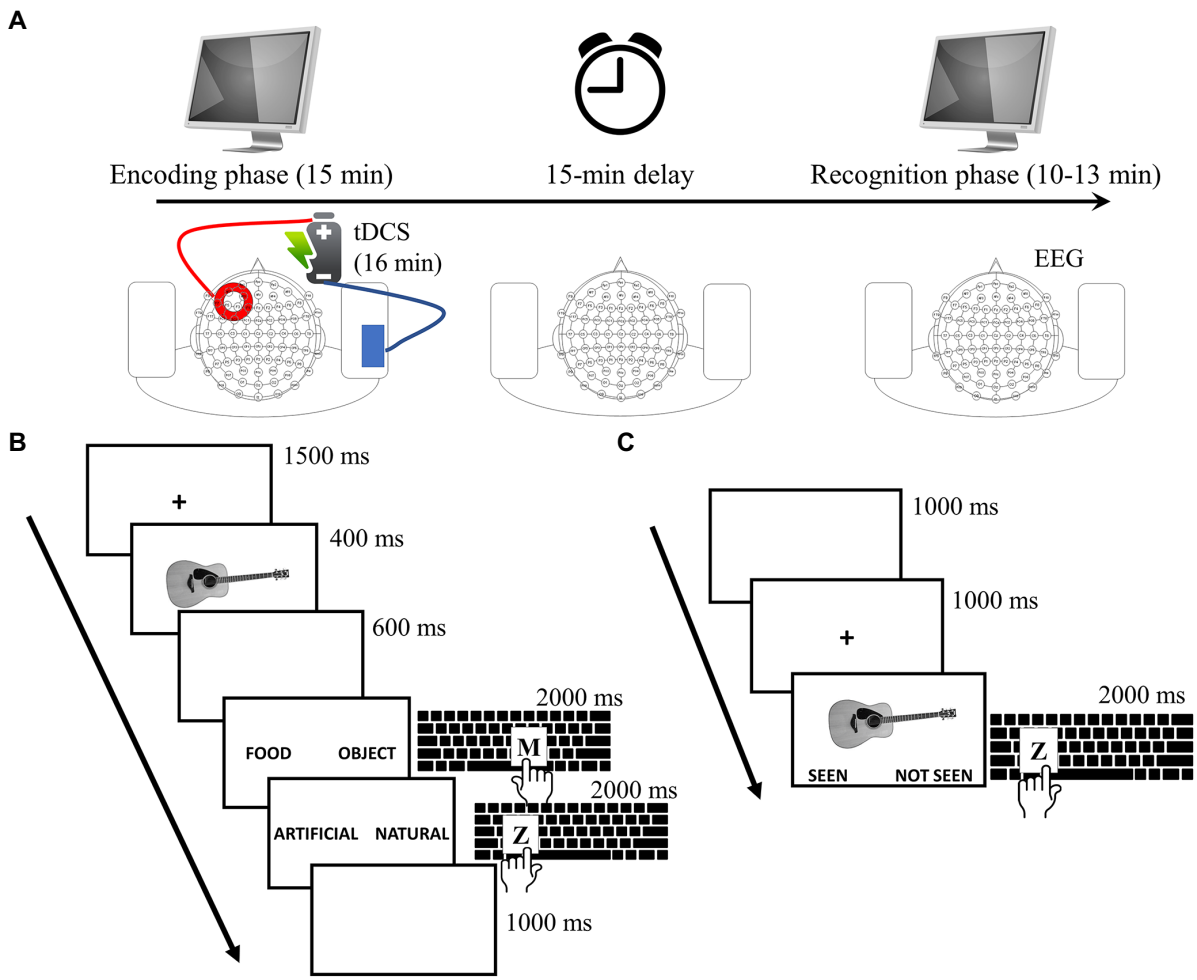
Experimental procedures. **(A)** Anodal or sham tDCS was administered during the encoding phase of the episodic memory (EM) task. After a delay of 15 min, participants performed the recognition phase of the EM task during which EEG data were collected. **(B)** Trial procedure of the encoding phase of the EM task. In each trial, an image to be remembered is presented followed by two different semantic choices. The original task was performed in Italian. **(C)** Trial procedure of the recognition phase of the EM task. Participants are required to decide whether each image was old (already seen in the encoding phase) or new (not seen). The original task was performed in Italian.

The present study adopted a single-blind within-subjects sham-controlled experimental design, in which participants underwent two experimental sessions, at the same time of the day, on two different days separated by at least 1 week. This was done to reduce variability in the subject’s physiological state during the day and to ensure a full wash-out of the effects from one session to the other. In each session, participants performed an EM task consisting of two phases: an encoding phase and a retrieval phase of an Old/New recognition task. Either atDCS or sham tDCS in counterbalanced order was applied over the left DLPFC only during the encoding phase. Electroencephalographic (EEG) data were collected during the recognition phase. Participants were blinded to the stimulation they received. Figure 1A illustrates the experimental design.

### 2.3. Episodic memory task

The encoding phase and the subsequent recognition phase were separated by a delay of 15 min. During the encoding phase, 120 black-and-white images of common objects, food and animals selected from the Bank of Standardized Stimuli (BOSS; Brodeur et al., 2010) were presented in random order. Participants were instructed to memorize the images, as they would be asked to subsequently recognize them. Stimuli were presented in trials with the following structure: (1) 400 ms image presentation; (2) blank screen for 600 ms; (3) first semantic encoding for 2,000 ms; (4) second semantic encoding for 2,000 ms; and finally, (5) inter-trial-interval (ITI) of 7,500 ms. The semantic encoding of the task was controlled by asking participants to categorize images. For this purpose, participants were requested to make two subsequent choices after each image presentation by pressing two different keyboard buttons with their left or right index finger (spatially congruent to the side of the screen where the chosen word appeared). Hence, they should first indicate the category of the image (object, animal, food) and then whether the image represented a natural or an artificial item (they were instructed to consider those items that cannot exist without human intervention as artificial, while in the opposite case, they should be considered natural). Indicating whether food images represented a natural or an artificial item might have required a more careful choice as compared to object and animal images (which have a tendency to be almost exclusively artificial and natural, respectively) and may have potentially led to a better encoding. Nevertheless, the three image categories were equally represented in the two experimental sessions (i.e., sham, atDCS) thus not affecting the comparison of the results between sham and atDCS conditions.

During the recognition phase, the 120 images displayed during the encoding phase (old images) were presented randomly intermixed with 120 new images. Participants were instructed to indicate whether the image was “old” (i.e., already seen in the encoding phase) or “new” (i.e., not seen in the encoding phase) by pressing two different keyboard buttons with their left or right index finger (spatially congruent to the side of the screen where the chosen response appeared, i.e., already seen/not seen). Each image remained on the screen for 2,000 ms or until the subject’s response (whichever happened first), which was followed by a 1,000 ms delay. The encoding and retrieval trial procedures are depicted in Figures 1B,C, respectively. Two sets containing different images (Figures 1A,B; with the three image categories equally represented in the two sets) were used for the two sessions (sham, anodal). The order of set presentation was counterbalanced across participants and across sessions.

Behavioral data were collected during the recognition phase. The percentage of Old pictures correctly classified as old (Hit rate), and the percentage of New pictures correctly classified as new (Correct Rejections rate, CR rate) were analyzed.

### 2.4. Transcranial direct current stimulation parameters

Anodal tDCS was delivered by a battery-driven stimulator (BrainStim, EMS, Bologna, Italy) through a pair of rubber electrodes for 16 min, starting approximately 1 min before the beginning of the encoding phase and lasting for its entire duration. The intensity of the stimulation was 1.5 mA. The anode ring electrode (5 cm diameter, area 14.72 cm^2^ current density 0.1 mA/cm^2^) was placed below the EEG cap surrounding the F3 electrode (International 10–20 EEG System), and Ten20 conductive paste (Weaver and Company, Aurora, CO, United States) was used to obtain a perfect adherence to the head. The cathode/return electrode (5 cm × 9.5 cm, area 47.5 cm^2^, current density 0.03 mA/cm^2^) was located on the right shoulder by elastic bands. Impedance levels were below 5 kΩ. In the sham condition, the parameters were the same with the exception that the current was turned off 20 s after the stimulation began and turned on again during the final 20 s of the task. Safety procedures were adopted based on non-invasive brain stimulation approaches (Antal et al., 2017). Immediately after the end of each experimental session, participants completed a standardized questionnaire assessing the sensations induced by tDCS (Fertonani et al., 2015; Antal et al., 2017). Participants were required to evaluate the intensity of several sensations (i.e., itching/irritation, pain, burning, heat, iron taste, fatigue) through a 5-point scale (0 = none; 1 = mild; 2 = moderate; 3 = considerable; 4 = strong). Furthermore, at the end of the last experimental session (i.e., at the end of their participation in the whole protocol), participants were asked to guess whether they received real or “placebo” (i.e., sham) stimulation in each of the two sessions. For this, they were required to select one response (“Real stimulation,” “Sham,” or “I do not know”) for each experimental session.

### 2.5. EEG recording and processing

The EEG was recorded *via* 60 sintered Ag/AgCl electrodes placed in an elastic cap (EasyCap, GmbH, Herrsching, Germany) according to the International 10–10 System. All electrodes were referenced online to the right mastoid, and the FPz electrode served as the ground. The horizontal electrooculogram (EOG) was recorded *via* two electrodes placed at the outer canthi of both eyes, whereas the vertical EOG was recorded *via* two electrodes placed supra-and infraorbitally to the right eye. The EEG recorded with a 16-bit DC amplifier (BrainAmp Brain Products GmbH, Herrsching, Germany) was continuously digitized at a rate of 5,000 Hz (online bandpass filter 0.01–1,000 Hz), and the electrode impedance was maintained below 10 kΩ. Only the signal recorded during the recognition phase of the task was considered for the present study. In the offline processing of the signal, the continuous EEG and EOG signals were downsampled to 500 Hz, and the EEG was rereferenced to the left mastoid electrode. Then, a digital bandpass filter was applied (0.1–40 Hz; 12 dB/octave slope), and ocular artifacts were corrected by running independent component analysis and visually selecting artifactual components (Infomax algorithm, implemented in Brain Vision Analyzer; Lee et al., 1999).

With the aim of evaluating the ERP components of interest, the EEG was segmented into stimulus-locked epochs of 2,200 ms (200 pre-stimulus). The epochs including Hits (Old images recognized as old, i.e., “Old” condition) and Correct Rejections (New images recognized as new, i.e., “New” condition) were evaluated. All epochs were corrected to the mean voltage of the first 200 ms of each epoch (pre-stimulus baseline), and segments exceeding ±100 μV were automatically rejected. The epochs were then averaged separately for the Old and New conditions, and a minimum of 41 artifact-free epochs were averaged for each condition. Finally, to obtain the Old-New difference waveforms, the averaged epochs related to the “new” trials were subtracted from the averaged epochs related to the “old” trials (Friedman, 2013). All the offline processing of the signal was performed using Brain Vision Analyzer 2.2.

The mean amplitude (in μV) of the Early Frontal (EF; in the 400–700 ms interval at the F3, Fz, and F4 electrodes), Parietal (P; in the 450–800 ms interval at P3, Pz, and P4) and Late Frontal (LF; in the 800–1,600 ms interval at F3, Fz, and F4) Old-New effects were evaluated in the Old-New difference waveforms (Gutchess et al., 2007; Nessler et al., 2007; Guillaume et al., 2009; Wolk et al., 2009; Friedman, 2013). Since previous ERP studies indicated that Old-New EM effects occur later in aging and as done in previous studies (see, for example, Guillaume et al., 2009 for EF), later and longer intervals than those used in studies on younger samples were used to capture these effects for both groups.

### 2.6. Statistical analysis

To investigate baseline differences between the middle-aged and older adults, demographic factors and cognitive function assessments were analyzed by independent sample *t*-test and Chi-squared test for continuous variables and dichotomous variables, respectively (see Table 1).

To investigate the effect of tDCS on recognition performance in the two age groups, two separate repeated measures ANCOVAs were conducted on the Hit and CR rates with “Session” (anodal, sham) as a within-subjects factor, “Group” (middle-aged, older adults) as a between-subjects factor and education as a covariate. To investigate the effect of tDCS on the neural correlates of EM, mean amplitude values of the ERP Old-New effect components (EF, P, LF) were submitted to separated repeated measures ANCOVAs with “Session” (anodal, sham) and “Electrode” (F3, Fz, F4 for EF and LF; P3, Pz, P4 for P) as within-subjects factors, “Group” (middle-aged, older adults) as a between-subjects factor and education as a covariate. *p-*values ≤0.05 were considered significant. *Post hoc* comparisons were performed with Bonferroni correction for multiple comparisons. Partial eta squared values (*η_p_*^2^) are reported as estimates of effect size (Richardson, 2011), while critical F and 1-β scores are reported as power measures for each significant ANCOVA effect.

Data related to the sensations induced by tDCS were analyzed with a non-parametric Wilcoxon test to compare the different session (anodal, sham) in the two groups of participants (middle-aged, older adults). Statistical analyses were performed with IBM SPSS Statistics, and statistical power and analyses were estimated using GPower 3.1 (Faul et al., 2009).

## 3. Results

The only two significant differences among cognitive and demographical variables of the two groups were age (*p* < 0.001) and years of education (*p* < 0.01), with middle-aged adults being younger and showing a higher degree of education (Table 1).

Participants were unable to discriminate between the two tDCS conditions (anodal, sham). Over 36 participants, 25 responded they received “Real stimulation” in both sessions, 3 responded that they received “Sham” stimulation in both sessions, 7 selected “I do not know.” Only one participant was able to correctly deduct the stimulation received in both sessions.

Separated analysis performed on data collected in middle-aged and older adults showed no differences between anodal and sham tDCS on irritation, burning, heat, iron taste and fatigue sensations (middle-aged all *p* > 0.26; older adults all *p* > 0.18). Therefore, atDCS was indistinguishable from the sham tDCS condition in all the sensations; hence, the blinding between the two stimulation conditions reduced experimental biases on participants’ expectations (see Supplementary material for the data).

### 3.1. Behavioral results

**Figure 2:**
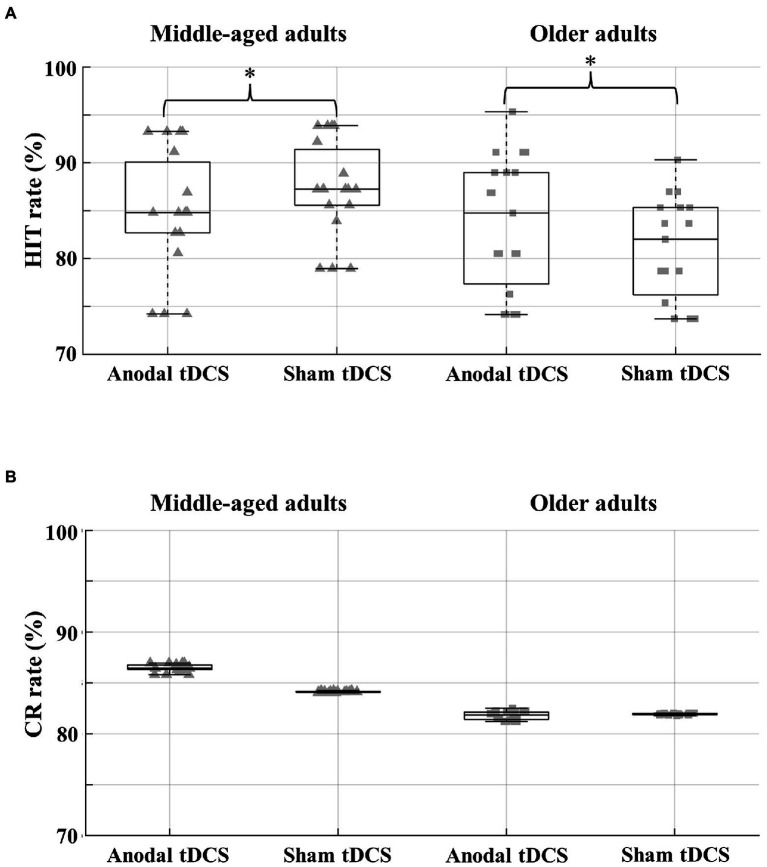
Behavioral results. **(A)** Boxplots depicting the hit rate obtained during the recognition EM task in middle-aged (left) and older adults (right) after anodal and sham tDCS sessions; **(B)** Boxplots depicting the correct rejection rate obtained during the recognition EM task in middle-aged (left) and older adults (right) after anodal and sham tDCS sessions. For each boxplot, the rectangles represent the interquartile range from the 25th percentile (quartile 1, Q1; lower black bar) to the 75th percentile (quartile 3, Q3; upper black bar), with the horizontal bar inside representing the 50th percentile (quartile 2, Q2, median), and the horizontal bars depicting the largest (upper bar) and smallest (lower bar) values within 1.5 times the interquartile range above the 75th percentile and below the 25th percentile, respectively. * indicates a significant difference.

ANCOVA results on the Hit rates showed a significant Group × Session interaction [*F*(1,35) = 7.51, *p* = 0.01, *η_p_*^2^ = 0.177, critical *F* = 4.12, 1−β = 0.79]. *Post hoc* comparisons revealed that middle-aged subjects showed a detrimental effect on recognition performance after receiving anodal tDCS, with significantly lower Hit rate values (mean = 84.9, SD = 10.1) than after sham tDCS (*p* = 0.043; mean = 87.3, SD = 9.7). In contrast, recognition performance in the older adults was ameliorated after anodal tDCS, with a significantly higher hit rate (*p* = 0.048; mean = 83.6, SD = 13.4) than after sham tDCS (mean = 81.1, SD = 14.2) (Figure 2A). No significant differences emerged when comparing recognition performance (Hit rate) between the two age groups (*p* > 0.10). No significant main effect or interaction emerged when considering CR rates as the dependent variable (Figure 2B).

### 3.2. Electrophysiological results

**Figure 3:**
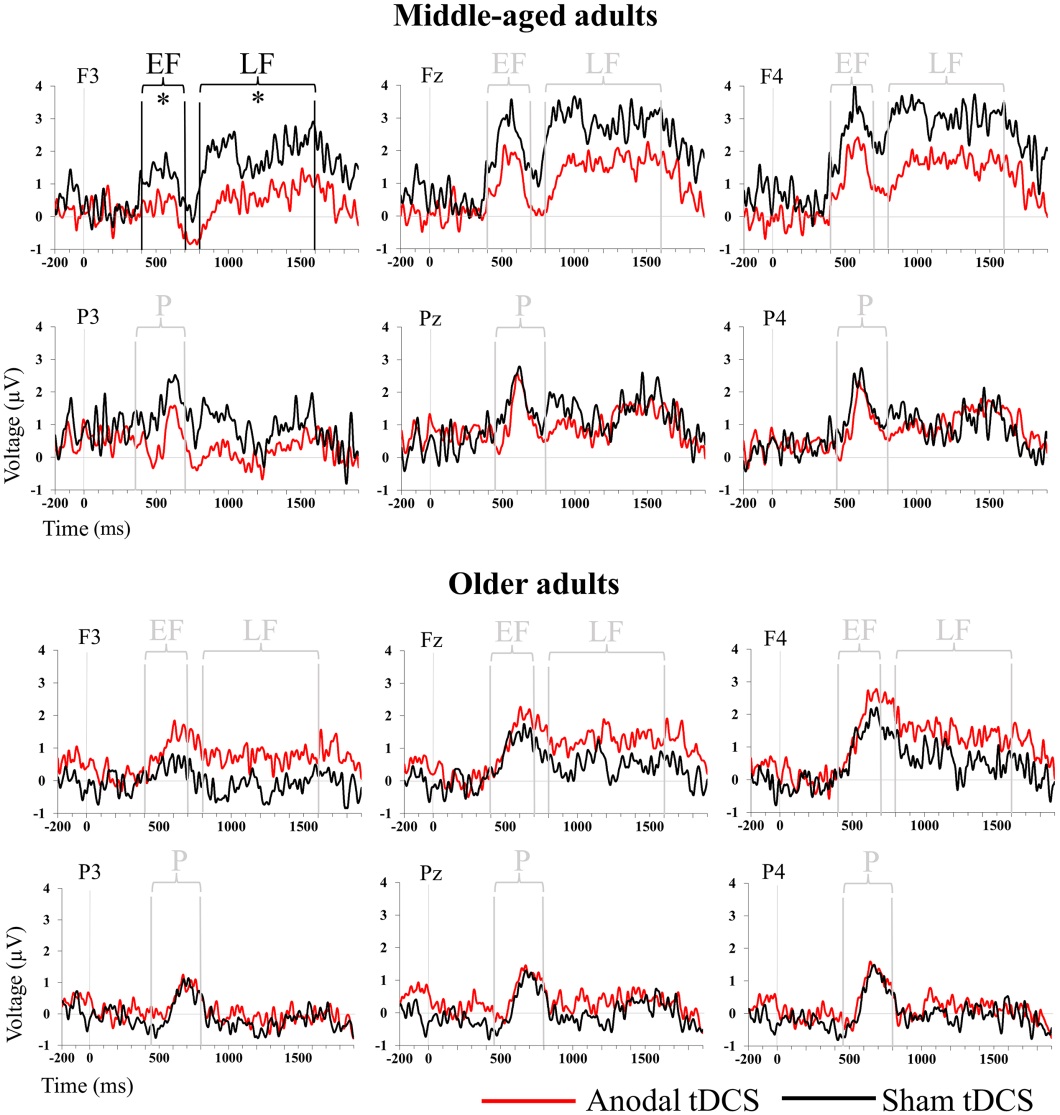
Grand-average ERP waveforms elicited by the old stimuli (hit) in the Anodal (red line) and Sham (black line) sessions for the middle-aged (upper panel) and older (lower panel) adults at the F3, Fz, F4, P3, Pz, and P4 electrodes. Vertical lines highlight the time window considered for the different ERP components. Black vertical lines with an asterisk indicate statistically significant differences between the anodal and sham conditions. EF, Early Frontal Old-New effect; LF, Late Frontal Old-New effect; P, Parietal Old-New effect. * indicates a significant difference.

Figure 3 depicts grand-averaged ERP waveforms during the recognition task for Hit responses after anodal and sham tDCS in the middle-aged and older adult groups. Mean amplitude values of EF, P, and LF old/new EM effect components are reported in Table 2.

**Table 2:** Mean amplitudes and standard deviations (in parentheses) of the Early Frontal (EF), Parietal (P), and Late Frontal (LF) effects, measured in the Old-New difference waveforms, in the anodal and sham sessions, for the middle-aged adults and older adults at each electrode position where the effect was evaluated (F3, Fz, and F4 for the EF and LF effects, and P3, Pz, and P4 for the P effect).

		Early frontal effect (400–700 ms)		Parietal effect (450–800 ms)		Late frontal effect (800–1,600 ms)	
		Anodal	Sham	Anodal	Sham	Anodal	Sham
F3/P3	Middle-aged adults	−0.21 (2.27)	0.61 (1.48)	−0.12 (1.85)	0.63 (2.28)	0.22 (2.29)	1.50 (3.21)
	Older adults	0.61 (0.97)	0.42 (1.41)	0.09 (1.04)	0.18 (1.76)	0.11 (1.86)	0.40 (1.56)
Fz/Pz	Middle-aged adults	1.02 (1.89)	1.40 (1.53)	0.38 (1.90)	1.22 (2.89)	1.35 (2.05)	1.85 (2.26)
	Older adults	1.09 (1.19)	1.12 (1.45)	0.03 (1.45)	0.36 (2.12)	0.81 (1.83)	0.20 (1.38)
F4/P4	Middle-aged adults	1.52 (1.52)	1.81 (1.60)	0.72 (1.77)	1.30 (2.33)	1.59 (1.87)	2.04 (2.52)
	Older adults	1.51 (1.12)	1.51 (1.43)	0.37 (1.54)	0.60 (1.62)	0.97 (1.75)	0.45 (1.58)

#### 3.2.1. Early frontal old-new effect

ANCOVA results on EF mean amplitude revealed significant main effects of “Session” [*F*(1,35) = 4.20, *p* = 0.048, *η_p_*^2^ = 0.107, critical *F* = 4.12, 1−β = 0.55] and “Electrode” [*F*(2,70) = 4.99, *p* = 0.023, *η_p_*^2^ = 0.125, critical F = 4.12, 1−β = 0.62], together with a significant “Group × Session × Electrode” interaction [*F*(2,70) = 3.57, *p* = 0.033, *η_p_*^2^ = 0.093, critical *F* = 4.12, 1-β = 0.48]. When considering the covariate (i.e., education), the “Session” factor was found to be non-significant (*p* = 0.46). *Post hoc* comparisons for the “Electrode” main effect revealed that EF amplitude was significantly greater in the right hemisphere (F4; mean = 1.59, SEM = 0.18) than in both the midline (Fz; mean = 1.16, SEM = 0.19) and left hemisphere (F3; mean = 0.36, SEM = 0.20) (all *p* < 0.001).

*Post hoc* comparisons to disentangle the significant “Group × Session × Electrode” interaction revealed that middle-aged participants showed a significant reduction in left hemisphere EF amplitude after anodal (F3: mean = −0.54, SEM = 0.42) compared to sham (F3: mean = 0.67, SEM = 0.36) (*p* = 0.020) tDCS. Additionally, after anodal tDCS, middle-aged participants displayed a significantly smaller (*p* = 0.024) left frontal (F3) EF amplitude (F3: mean = −0.54, SEM = 0.42) than older adults (F3: mean = 0.94, SEM = 0.42).

No difference emerged when comparing the two groups after sham stimulation (*p* > 0.48).

#### 3.2.2. Parietal old-new effect

ANCOVA results of the P old/new effect mean amplitude did not reveal any significant main effect or significant interaction (all *p* > 0.12).

#### 3.2.3. Late frontal old-new effect

The results of the LF mean amplitude revealed a significant “Session × Electrode” interaction [*F*(2,70) = 4.96, *p* = 0.01, *η_p_*^2^ = 0.124, critical *F* = 4.12, 1−β = 0.62] and a significant “Group × Session × Electrode” interaction [*F*(2,70) = 4.36, *p* = 0.016, *η_p_*^2^ = 0.111, critical *F* = 4.12, 1−β = 0.56]. *Post hoc* comparisons to disentangle the significant “Group × Session × Electrode” interaction revealed that middle-aged participants showed a reduction in LF amplitude over the left hemisphere after anodal (F3: mean = −0.12, SEM = 0.50) compared to sham (F3: mean = 1.55, SEM = 0.64) (*p* = 0.023) tDCS. Comparing the LF effect between the two groups of participants, *post hoc* analysis revealed that older adults after sham (*p* = 0.046) showed a reduction in frontal (F3) LF amplitude (compared to middle-aged) but not after anodal tDCS (*p* = 0.466).

## 4. Discussion

In the present study, we investigated the behavioral and neural effects of atDCS applied over the left DLPFC during the encoding phase of an EM Old-New recognition task in healthy middle-aged and older adults. Behavioral results showed that age modulated the effect of atDCS during the encoding on EM abilities, with an opposite pattern of results in the two age groups: whereas older adults improved their recognition performance, middle-aged participants showed a worsening in performance. This is the first study to highlight such an age-related difference in atDCS outcomes regarding memory tasks.

Present results on how tDCS applied during the encoding phase of an EM task differently affected the behavioral indexes in the two age groups may help to disentangle the specific mechanisms of action of tDCS upon EM processes. Indeed, a better/worse recognition performance can rely either on a more/less efficient encoding of target images (Old stimuli) or on a reduction/increase of interference of distractor stimuli (New stimuli) during the recognition phase. The present results showed that Hits (Old stimuli correctly judged as old) were enhanced by 2.50% and reduced by 2.41% after atDCS (compared to sham) in older and middle-aged adults, respectively. In contrast, CR (New stimuli correctly judged as new) were not affected by atDCS. Thus, we hypothesize that prefrontal atDCS affected recognition memory performance by strengthening (or decreasing) the memory traces of old to-be-remembered images (those presented during the delivery of tDCS) rather than by acting upon the response to distractor stimuli (New items) that have not been presented during the encoding phase. These findings appear consistent with an “online,” within-session effect of atDCS, rather than an “offline” post-tDCS session improvement (Sandrini et al., 2019), emphasizing that task component and age, not only tDCS, are relevant elements in determining the final effect.

Electrophysiological results showed that the behavioral deterioration observed in the group of middle-aged adults was mirrored with a modulation of specific EM neural correlates. Middle-aged adults showed a significant reduction in the left hemisphere EF and LF components after atDCS but not after sham tDCS. In contrast, the behavioral improvement observed in the group of older adults was not associated with any statistically significant modification of the electrophysiological components. To date, no study has investigated the effects of tDCS on these ERP signatures of EM in older adults.

It is widely held that recognition memory is supported by two functionally distinct processes, namely, familiarity and recollection (Friedman, 2013). The present ERP results showed that the first EM component affected by atDCS in the group of middle-aged adults was the EF effect, which represents a putative mechanism indexing familiarity-based recognition processes (Curran, 2000; Rugg and Curran, 2007). Through the reduction in EF amplitude in middle-aged adults, atDCS might have interfered with mnemonic processes relying on familiarity (i.e., fast and relatively automatic processes based on feeling that an event is old or new in the absence of confirmatory contextual information; Daselaar et al., 2006), causing a reduction in recognition efficiency in this age group.

Together with familiarity process modulation, the ERP results showed that atDCS also induced a reduction in the LF effect amplitude in middle-aged adults only in the stimulated left DLPFC. This later positive component is usually associated with post-decisional monitoring aspects of mnemonic processes more linked to executive functioning (Friedman and Johnson, 2000; Rugg, 2004; Hayama et al., 2008). However, atDCS over the left DLPFC did not induce any modulation of the recollection-based mnemonic processes, which are indexed by the parietal EM.

To our knowledge, there is only one other study that analyzed the effects of atDCS on ERP components elicited in an EM task (Lu et al., 2015). This study found modulations produced by atDCS in healthy young participants in the N400 and P600 ERP components, observable in the direct traces in a verbal EM task. The amplitude of N400 was enhanced in frontal regions and decreased in parietal regions after atDCS, while P600 amplitude was enhanced in parietal locations. However, the author do not discuss the functional significance of these modulations, nor the possible modulations over the old-new effects.

Interestingly, significant ERP amplitude reductions were restricted to the stimulated region (i.e., left prefrontal cortex) in the present results. The spatial specificity of this effect also aligns well with the hypothesized mechanism of action of atDCS on the behavioral measures (see above). In fact, there is evidence indicating that tDCS modulates neuro-metabolite levels at the site of stimulation and that this translates into alterations in behavioral outcomes (Chhabra et al., 2021). Notably, the “online” obtained effect on encoding processes is in accordance with the HERA (Hemispheric Encoding Retrieval Asymmetry) model, which postulates that the left DLPFC is crucially involved in the encoding of memory contents, whereas the right DLPFC is crucial for retrieval (Tulving et al., 1994; Nyberg et al., 1996; Habib et al., 2003; Manenti et al., 2012).

The opposite pattern of tDCS effects in middle-aged and older adults may point toward differential functionality of the system, highlighting the importance of the interaction of brain stimulation with brain activity in defining the behavioral outcome (Miniussi et al., 2010, 2013). Among the possible explanations put forward in the literature to unfold the inter-individual differences in tDCS efficacy, the present results may fit with previous studies supporting the hypothesis that tDCS gains are restricted to higher task demands (e.g., Li et al., 2015). Accordingly, older adults might experience a higher cognitive load than middle-aged adults when performing the same EM task and thus might benefit from atDCS-induced effects. However, cognitive load was not measured in this study, and no between-group difference in recognition performance was observed between groups after sham stimulation. Previous studies also suggested graded beneficial effects of tDCS from healthy young adults to physiological aging and then to pathological aging, with low cognitive performance groups benefitting more from the stimulation and with possible deleterious outcomes in healthy young adults who do not present a need for stimulation (Hsu et al., 2015). Although this framework appears promising for the interpretation of the present results, again, we did not observe any differences between middle-aged and older adults in cognitive functioning.

Another model that has been proposed refers to the “stochastic resonance,” which states that the same amount of stimulation may induce different ratios of signal-to-noise increase depending on the subject’s activation state (Miniussi et al., 2013; Fertonani and Miniussi, 2017). Consequently, the distinct neural activation that older adults might need to execute the task might have represented the optimal state to shape the beneficial behavioral outcome. Furthermore, it has been proposed that the leeway for tDCS modulation depends on the dissociation of the neural system from a previous optimal state. Therefore, whereas middle-aged adults might function close to their homoeostatic optimum (with a relatively small margin for tDCS improvement), older adults might shift away from it, possibly making their brain more amenable to tDCS-induced gains (Habich et al., 2020a,b).

However, in the absence of imaging data characterizing the neural state of the two groups of participants, it is difficult to draw a broader conclusion about the neural basis underlying the opposite tDCS effects.

Although the present results appear promising, some issues need to be addressed. First, the limitations of the cross-sectional designs need to be considered when interpreting the present results. A longitudinal study assessing the within-subjects progression along the aging lifespan is needed to verify how the impact of tDCS on EM changes as the aging process advances. Another limitation that needs to be addressed regards the lack of a control condition, such a control target site and/or different intensities. This type of control conditions are important to ensure that changes in memory performance are specific to the protocol adopted. However, our study did not aimed at investigating the optimal tDCS protocol to induce memory improvements but to assess whether the same protocol applied in different aging stages induced differential effects. We want to emphasize that it remains to be clarified whether our results generalize to other tDCS electrode configurations and stimulation parameters, highlighting the importance and non-triviality of replicating and extending our observations while varying methodological factors.

In our study, we did not have MRI scans of the participants, thus impeding us from controlling for whether different levels of cortical atrophy between middle-aged and older adults may have been associated with different electric field characteristics.

Our experimental design did not include a baseline assessment in the EM task, like in several other paradigms where the main aim was to assess tDCS effects on memory in two different groups (e.g., Manenti et al., 2013; Brambilla et al., 2015; Fiori et al., 2017; Leach et al., 2019). The inclusion of a baseline would have augmented the length of experimental sessions, with possible detrimental effects, e.g., increasing fatigue level and/or decreasing participants’ adherence to the project. Importantly, we did not find differences between groups in any of the behavioral and ERP variables in the sham condition nor in any of the neuropsychological measures at baseline. Moreover, we ensured that experimental conditions were exactly the same in both experimental sessions except for the tDCS condition. Hence, although group differences at baseline in this specific EM task cannot be completely ruled out, it is likely that behavioral and neural differences are due to the application of atDCS.

Here, we provide evidence for an age-dependent effect of atDCS over the DLPFC on EM and its underlying electrophysiological substrates, indicating different and opposing modulatory patterns along the healthy aging continuum. These results contribute to a better understanding of the differential age effects of tDCS, giving support to the notion that tDCS combined with a task can be used to improve cognition only under given conditions. Because of altered neuroplasticity and network dynamics with age, a detailed investigation of older adults covering a wide age range is of paramount importance. Such an investigation would both elucidate the complexity of tDCS effects at the neurophysiological level and help develop more individually tailored interventional protocols in healthy populations and in age-related diseases, such as Alzheimer’s disease and dementia.

## Data availability statement

The original contributions presented in the study are publicly available. This data can be found here: https://gin.g-node.org/MartaBortoletto/Bagattini_Cid-Fernandex_et_al_2023.

## Ethics statement

The studies involving human participants were reviewed and approved by Ethics Committee of the IRCCS Centro San Giovanni di Dio Fatebenefratelli (*Via* Pilastroni, 4 Brescia – Italy). The patients/participants provided their written informed consent to participate in this study.

## Author contributions

CB: conceptualization, methodology, investigation, formal analysis, data curation, writing – original draft, writing – review and editing, visualization, and project administration. SC-F: methodology, investigation, software, formal analysis, writing – original draft, writing – review and editing, and visualization. MBu: formal analysis and writing – review and editing. CM: conceptualization, methodology, writing – review and editing, supervision, and funding acquisition. MBo: conceptualization, methodology, writing – review and editing, project administration, and supervision. All authors contributed to the article and approved the submitted version.

## Funding

This study was funded by the Italian Ministry of Health Ricerca Finalizzata to CM (RF-2013-02356444) and Ricerca Corrente. SC-F was founded by Galician Postdoctoral Grants Plan (ED481B 2016/078-0, Xunta de Galicia).

## Conflict of interest

The authors declare that the research was conducted in the absence of any commercial or financial relationships that could be construed as a potential conflict of interest.

## Publisher’s note

All claims expressed in this article are solely those of the authors and do not necessarily represent those of their affiliated organizations, or those of the publisher, the editors and the reviewers. Any product that may be evaluated in this article, or claim that may be made by its manufacturer, is not guaranteed or endorsed by the publisher.
