# Supplementary material for: Opposite pattern of transcranial direct current stimulation effects in middle-aged and older adults: Behavioral and neurophysiological evidence
Source: Front Aging Neurosci. 2023 Jan 25;15:1087749. doi: 10.3389/fnagi.2023.1087749 (PMC9905246; doi:10.3389/fnagi.2023.1087749)
Supplement: Supplementary file 1 [file Table_1.docx]

|  | **Irritation** | **Pain** | **Burning** | **Heat** | **Iron taste** | **Fatigue** |
| --- | --- | --- | --- | --- | --- | --- |
| **Anodal** | 1.06 | 0.17 | 0.44 | 0.29 | 0.00 | 0.06 |
| **Sham** | 0.82 | 0.24 | 0.41 | 0.06 | 0.00 | 0.06 |
| ***p-value*** | 0.366 | 0.705 | 0.666 | 0.257 | 1.000 | 1.000 |

**Supplementary material**

**Supplementary Table 1.** Mean intensity of the sensations reported after anodal and sham tDCS by *middle-aged* participants. The sensation intensity is presented on a 5-point scale as follows: 0 = None; 1 = Mild; 2 = Moderate; 3 = Considerable; 4 = Strong.

**Supplementary Table 2.** Mean intensity of the sensations reported after anodal and sham tDCS by *older* participants. The sensation intensity is presented on a 5-point scale as follows: 0 = None; 1 = Mild; 2 = Moderate; 3 = Considerable; 4 = Strong.

|  | **Irritation** | **Pain** | **Burning** | **Heat** | **Iron taste** | **Fatigue** |
| --- | --- | --- | --- | --- | --- | --- |
| **Anodal** | 1.06 | 0.11 | 0.61 | 0.06 | 0.00 | 0.00 |
| **Sham** | 0.83 | 0.28 | 0.50 | 0.11 | 0.06 | 0.00 |
| ***p-value*** | 0.285 | 0.180 | 0.458 | 0.655 | 0.317 | 1.000 |

**Supplementary Table 3.** F-values from the two-factor ANCOVAs (Session x Group) for Hit and CR rates, and the three-factor ANCOVAs (Session x Group x Electrode) for the EF, LF and P effects.

| **ANCOVA (S x G)** | **Hit rate** | | **CR rate** | |
| --- | --- | --- | --- | --- |
| S | 1.661  df: 1/35  p = 0.206 | | 0.022  df: 1/35  p = 0.884 | |
| G | 0.637  df: 1/35  p = 0.430 | | 0.8  df: 1/35  p = 0.377 | |
| S x G | **7.514****  df: 1/35  p = 0.01 | | 0.619  df: 1/35  p = 0.437 | |
| **ANCOVA (S x G x E)** | **EF effect** | **P effect** | | **LF effect** |
| S | **4.198***  df: 1/35  p = 0.048 | 0.283  df: 1/35  p = 0.598 | | 0.682  df: 1/35  p = 0.414 |
| G | 0.424  df: 1/35  p = 0.519 | 0.017  df: 1/35  p = 0.897 | | 1.536  df: 1/35  p = 0.223 |
| E | 4.991  df: 2/70  p = 0.023 | 2.222  df: 2/70  p = 0.136 | | 0.892  df: 2/70  p = 0.378 |
| S x G | 3.155  df: 1/35  p = 0.084 | 0.511  df: 1/35  p = 0.479 | | 3.577  df: 1/35  p = 0.067 |
| S x E | 0.805  df: 2/70  p = 0.418 | 0.331  df: 2/70  p = 0.719 | | **4.955***  df: 2/70  p = 0.018 |
| G x E | 2.123  df: 2/70  p = 0.127 | 0.198  df: 2/70  p = 0.821 | | 0.055  df: 2/70  p = 0.946 |
| S x G x E | **3.571***  df: 2/70  p = 0.033 | 0.457  df: 2/70  p = 0.635 | | **4.356***  df: 2/70  p = 0.016 |

**p ≤ 0.01, *p ≤ 0.05. S: Session factor, G: Group factor, S: Session factor, df: degrees of freedom.
